# Physicochemical, Textural, and Sensorial Properties of Soy Yogurt as Affected by Addition of Low Acyl Gellan Gum

**DOI:** 10.3390/gels8070453

**Published:** 2022-07-20

**Authors:** Xiao Kong, Ziqun Xiao, Mengdi Du, Kuaitian Wang, Wei Yu, Yuhang Chen, Zhili Liu, Yongqiang Cheng, Jing Gan

**Affiliations:** 1 College of Life Science, Yantai University, Yantai 264000, China; kongxiao0608@163.com (X.K.); xiaozq1019@163.com (Z.X.); dmd19980612@163.com (M.D.); wangkuaitian@163.com (K.W.); anhuiyuw@163.com (W.Y.); cyhzzzz@163.com (Y.C.); liu12128116@163.com (Z.L.); 2 Beijing Key Laboratory of Functional Food from Plant Resources, College of Food Science and Nutritional Engineering, China Agricultural University, Beijing 100083, China

**Keywords:** soy yogurt, low acyl gellan gum, stability, texture, sensorial property

## Abstract

Soy yogurt is plant-based dairy of great nutritional interest that is widely accepted in developing countries as a milk alternative. Poor stability has been an urgent problem to solve of soy yogurt products over past several years. The present study aimed to construct multiple network composite gel by adding low acyl gellan gum (LAG) to improve the stability. The effect of addition of LAG on property of soy yogurt was investigated by determining water holding capacity, texture, rheology, particle size, and zeta potential. The results showed that water holding capacity was significantly higher than control. The soy yogurt with 0.1% LAG had a stable gel network with much gel strength and viscosity, and strengthened interaction between complex gel. The addition of LAG increased the particle size and decreased zeta potential. Furthermore, sensory properties were acceptable. Therefore, during industrial production, LAG could act as an appropriate stabilizer to inhibit poor body and bring more desirable sensory characteristics of soy yogurt.

## 1. Introduction

Soybeans (*Glycine max* L. Merr.) are one of the most important leguminous crops in the world and a source of various nutrients for human health including protein, unsaturated fatty acids, vitamins, fiber, and isoflavones [1,2]. Many researchers have demonstrated that nutrient compositions and contents depend on the processing such as enzymatic hydrolysis, ultrasound, germination, and fermentation [3,4,5,6]. Specifically, fermentation is extensively used in boosting nutritive profile owing to its inexpensive cost and availability. For example, soy yogurt, produced by bacterial fermentation of soy milk, has attracted attention in the plant-based yogurt alternatives market due to its unique nutritional values [7]. Recently, it has been reported that soy yogurt showed health benefits related to high antioxidant activity, reduction in the risk of diabetic diseases, and prevention against chronic inflammation [8,9,10]. However, the manufacture of soy yogurt remains challenging. This is largely related to the appearance and texture properties as such products generally have stability issues caused by whey separation [11].

The essential forces driving soy protein with a conservative structure are physical interactions such as hydrophobic interaction and hydrogen bonding [12]. As easily influenced by ion strength and pH, single protein gel tends to flocculation aggregation accompanied by a decrease in texture and water retention [13]. In addition, the existing limits of disorderly aggregate structure, weak gel strength, and low disulfide bond content result in rough taste and incompact structure in soy protein gel products [14]. Currently, fabricating protein-polysaccharide composite gel is generally accepted and a widely used method to improve the stability property of the gel system. Polysaccharides not only control texture and flavor release, but are also very helpful in promoting sensorial properties of products and gaining acceptance from consumers [15,16]. Celus et al. [17] reported that pectin acting as anionic polysaccharide has been adsorbed on the surface of the protein under electrostatic interaction to promote repulsion between proteins, thus improving stability of the system. Wang et al. [18] has demonstrated that mesona chinensis polysaccharide, which is abundant in hydroxyls and carboxyls promote soybean protein aggregation, resulting in better composite gel characteristics. Zhao [19] also suggested that CaSO_4_-induced soybean protein isolate gel supplemented with konjac gum displayed higher breaking shear stress by strengthening hydrogen bonds, significantly affecting gel strength and shear stress. However, each one of the stabilizers presents limitations to its use. For carrageenan, it possesses stability problems under acidity conditions, and formation of soybean protein granules during heating, while gelatin solidifies at 25 °C, which is restricted by cold preservation [20,21]. Additionally, the concentration of stabilizer used must be taken into consideration. Yan et al. investigated the influences of corn fiber gum on textural properties and microstructures of double-network hydrogels, and revealed desired textural characteristics with the concentration of stabilizer above 1%, below 2% at a certain pH value [22]. Therefore, choice of the proper hydrocolloid type and concentration is a crucial point in the control phenomenon of syneresis.

LAG is a category of linear anionic polysaccharides and widely used as a thickener and stabilizer in the food, pharmaceuticals, and cosmetics industry [23,24,25]. The key advantages of LAG are its high clarity, thermal stability, acid tolerance, and safety. Furthermore, they possess excellent gelling properties. A previous report has revealed that LAG molecules transfer from a random coil to a double helix configuration, which further forming stable three-dimensional network structure during the cooling process [26]. Ge et al. investigated the effects of gellan gum on the rheological and thermal properties of set yogurt and found that the use of gellan gum improved the yogurt’s textural and rheological properties and resulted in a tight and uniform protein network [27]. Kiani discussed the influence of gellan gum on the structure and stability of doogh (a traditional yogurt-based Iranian drink). A continuous network of casein-rich strands was observed due to electrostatic attachment of gellan to fragments of casein gel [28]. Although numerous studies are currently focused on the application of gellan gum in casein protein colloid as a gelling agent, to our knowledge, there are few published studies on soybean proteins.

Based on superior gel properties, stability, and nutritional values of LAG and soy yogurt, the main objectives of this work were to (1) fabricate a binary compound gel using LAG and soy yogurt as a raw material; and (2) explore the effects of different concentrations of LAG on physicochemical (water holding capacity, rheological properties, microstructure), textural, and sensorial properties of soy yogurt. In general, it is expected to provide technical support and theoretical guidance for upgrading soy yogurt products.

## 2. Materials and Methods

### 2.1. Material

Low acyl gellan gum was purchased from Sigma-Aldrich Company (St. Louis, MO, USA) with a purity of 98%. Soybeans were a gift from China Agriculture University (Beijing, China). Yogurt starter culture (*Lactobacillus plantarum*) was obtained from extracts of fermented pickled Chinese cabbage using the previously described method. The strain was precultured with a Mann Rogosa Sharp (MRS) broth culture medium.

### 2.2. Preparation of Soy Yogurt

Soybeans were weighed (150 g) and soaked in distilled water overnight at room temperature. Next, the mixture was blended using a mixer for 1 min and then strained to extract the soy milk. Samples were placed in glass jars and pasteurized using a pressure vapor sterilizer at 115 °C for 15 min to prevent lump formation. After cooling to temperature, all jars were treated with LAG solutions (0%, 0.01%, 0.025%, 0.05%, 0.075%, and 0.1% *w*/*w*). The LAG solution was prepared by dissolving LAG powder (1 g) into 100 mL of sterile water under a heating condition. Then, 10% *Lactobacillus plantarum* was incubated in soy milk at the level of 10^9^ CFU/mL at 37 °C for 12 h. The time was determined by the final pH (about 4.0). Then, yogurt samples were stored in a refrigerator at 4 °C.

### 2.3. Water Holding Capacity

The water holding capacity of soy yogurt was carried out according to the method of Buldo et al. [29]. After incubation for 1 day at 37 °C, soy yogurts (30 g) were transferred into centrifuge tubes and centrifuged at 4000× *g* for 10 min at 4 °C. The supernatant was collected and weighted, and WHC of SY was calculated using the following equation:

WHC% = (1 − M_0_/M_1_) × 100% (1)

where M_0_ and M_1_ are the weight of the residue after centrifugation and the weight of SY before centrifugation, respectively.

### 2.4. Texture Measurements

The hardness, chewiness, adhesiveness, cohesiveness, and springiness index of soy yogurt samples were determined using a TMS-Pro Texture Analyzer from Food Technology Corporation(Sterling, VA, USA). The probe was a 25.4-mm diameter acrylic cylinder. It moved at a velocity of 1 mm/s and a trigger force of 0.05 N. The samples were compressed to 50% of their original height in a double cycle. Three replicates were performed.

### 2.5. Rheological Behavior

The rheological measurements were performed using a controlled tension rheometer (TA Instruments, model AR 1500ex, New Castle, DE, USA) method of Cruz et al. [30]. Soy yogurt samples were loaded on the inset plate with 6 cm of diameter and a gap of 1 mm performing oscillatory assays. The applied strain was 1%, and the frequency sweep was in the 0.01 to 1 Hz range. The rheological parameters’ storage modulus (G′) and loss modulus (G″) were monitored at 5 ± 0.1 °C.

### 2.6. Composite Gel Property Measurement

The particle size and zeta potential were performed by dynamic light scattering using NanoBrook90 Plus Zeta (Brookhaven Instruments Corporation, Holtsville, NY, USA) at room temperature. Before measurement, all samples were diluted at 1:200 in distilled water. The effective diameter and zeta potential were automatically calculated by the instrument based on proper sample count rates.

### 2.7. Sensory Evaluation

Sensorial evaluation was performed using the industry standard (Sensory Evaluation Principle of Soy Yogurt). Panelists (10 people, 50% female and 50% male) were recruited from graduates, being told they were evaluating soy yogurt with different LAG addition in advance. Evaluated attributes were color, elasticity, aroma, chewiness, flavor, and appearance. Each panelist evaluated a 20 mL sample in a white plastic cup coded number randomly. All samples were evaluated in 3 times replication at 4 °C. Sensorial attributes score of <5 were considered as less appealing; scores of 5> by >9 were considered as a boundary on acceptability; scores of 10> by >15 reflect good sensorial quality.

### 2.8. Principal Component Analysis

Principal component analysis (PCA) is a widely used multivariate analysis technique that can be applied to reduce dimensions by data transformation showing their correlation and trend. PCA applied for soy yogurts were carried out using the following method. The evaluation forms were collected from panelists. Datasets of above stated sensory attributes were put in SPSS 16 software. Through orthogonal transformation, a group of variables that may be correlated are converted into linearly unrelated variables to reduce dimension while maintaining the characteristics that the variance contributes the most. In order to keep original data information integrity, it is essential to find a direction to make the variance of the data projected in this direction largest which contains the most information about the differences in the original data. Therefore, the largest variance value occurred in PC1, the secondary in PC2. PC1 and PC2 are referred to the first principal component factor (Principal Component 1) and the second principal component factor (Principal Component 2), respectively. The original data are projected on the plane formed by PC1 and PC2, and represented by two-dimensional data to achieve dimension reduction. The value represents the percentage of data information could be interpreted.

### 2.9. Statistical Analysis

All diagrams were drawn using Origin 8.6 software (OriginLab Corporation, Northampton, MA, USA), and structural formulas were processed by Chem Draw 7.0 software (Cambridgesoft Corporation, MA, USA). All indicators were determined at least triplicates, the data were processed by Excel 2010 software (Microsoft Corporation, Redmond, WA, USA). The significant differences were analyzed and evaluated using SPSS 16.0 software (IBM Corporation, Armonk, NY, USA) (*p* < 0.05).

## 3. Results

### 3.1. Water Holding Capacity

**Figure 1:**
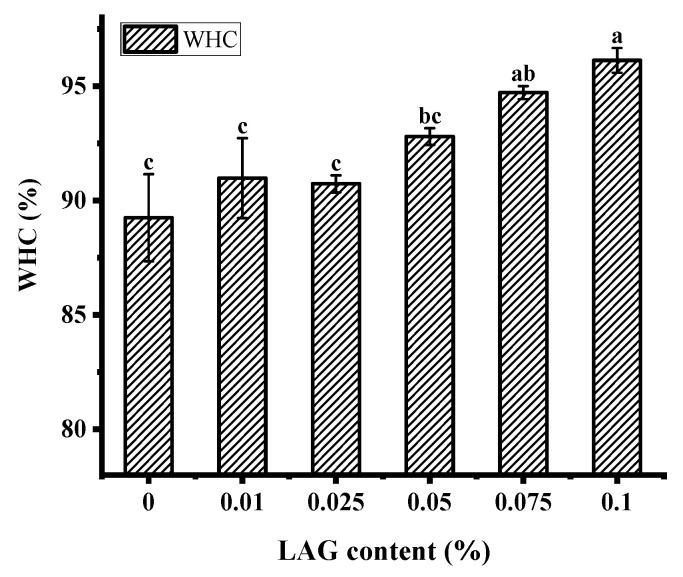
Water holding capacity of soy yogurt with different LAG contents. Different letters indicate that the values are significantly different (*p* < 0.001).

The water holding capacity (WHC) indicates the gel’s ability to retain the serum that is essential for a fermented product’s quality as it affects consumer acceptability [31]. As shown in Figure 1, with the LAG concentration increased, the WHC of soy yogurt showed a significant increase and then decrease. After this level, the WHC increased gradually from 90.75% to 96.13%. The overall results suggest that the absence of polysaccharides helped to improve soy protein gel system’s stability. Similar results were reported by Yan et al., which may be related to the cross-linked between polysaccharides and protein molecule formed denser and aggregated networks allowing composite gel to intercept water [32]. Furthermore, the structure of polysaccharide was abundant in hydrophilic groups that could enhance hydrogen bonding interaction to bind water [33].

### 3.2. Texture Profile Analysis

**Figure 2:**
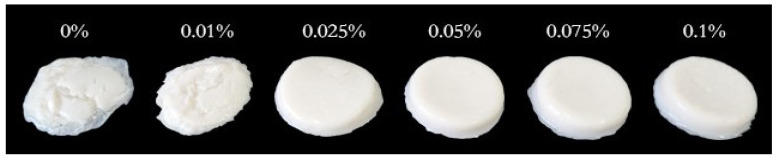
Images of soy yogurt with different LAG contents.

Textural profile analysis (TPA) is an important instrumental technique used to evaluate the textural properties of yogurt by imitating a mouth’s biting or chewing activity [33]. Table 1 shows the effect of LAG on the soy yogurt system’s six texture attributes: hardness (the force required to attain a certain deformation), adhesiveness (the effort needed for jaw movement in the mouth due to stickiness), cohesiveness (the level to which a material can be deformed before it is ruptured and is a measure of the strength of internal bonds), springiness (the extent to which the sample returns to its original height or volume after the deformation force is removed), gumminess (the energy required to chew solid foods into a state ready for swallowing), chewiness (the energy required to chew solid food). The hardness of soy yogurt added LAG was significantly higher than single protein gel, which might be associated with the higher WHC [27]. Furthermore, with the increase of LAG, an upward trend in adhesiveness, springiness, gumminess, and chewiness was observed. Overall, the addition of LAG at 0.1% resulted in superior texture characteristics. This is further supported by the images (Figure 2), which showed that the mixed gel could be much harder and springier, with the gelatin state being stronger. The possible reasons were as follows: Xu et al. reported that the increase in polysaccharides concentration in a mixed gel system promotes covalent cross-linking between molecules, which enhances the stiffness of a polysaccharide hydrogel network, causing stronger mechanical properties [34].

### 3.3. Rheological Studies

**Figure 3:**
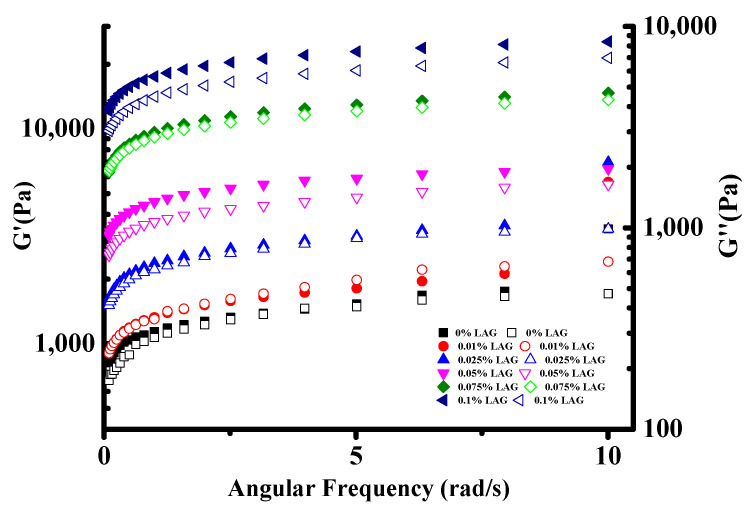
Frequency dependence of storage modulus G′ and loss modulus G″ for soy yogurt with different LAG contents.

The soy yogurt exhibited a smaller linear viscoelastic ranging from 0.25% to a 2.78% strain (Figures S1 and S2). Under 3% strain, the elastic modulus began to decrease. Therefore, the relationships of G′ and G″ with angular frequency were studied when the strain was controlled at 1%. As shown in Figure 3, an increasing trend in G′ and G″ value with the addition of LAG increased was observed in all soy yogurt samples, suggesting that LAG could yield more acid, which was beneficial for strengthening a mixed gel structure [33]. The larger the storage modulus, the denser the gel network with more interactions [35]. In addition, it is worth noting that, in all cases, the G′ values were greater than G″, which indicated that the soy yogurt exhibited a “solid-like” structure. These results suggest that the mixed gel systems had a high structural strength.

### 3.4. Properties of Complex Gel

**Figure 4:**
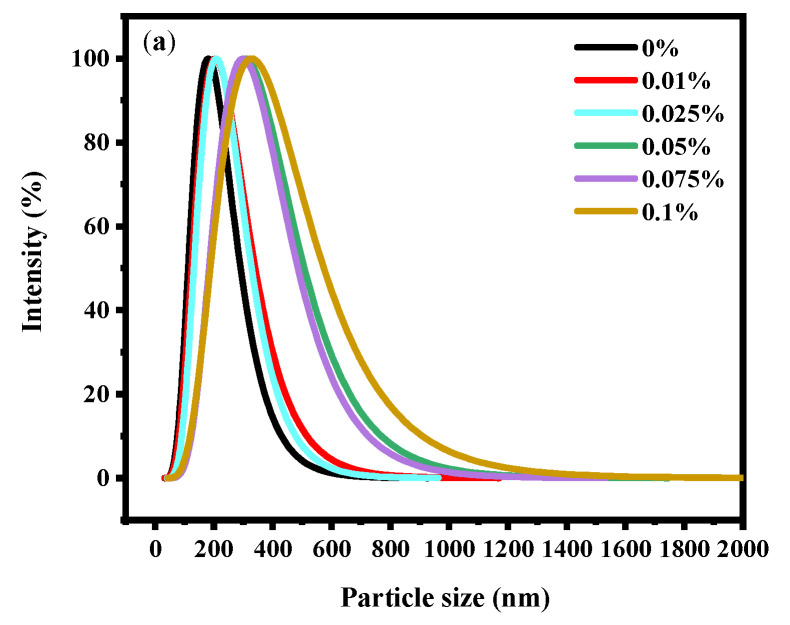

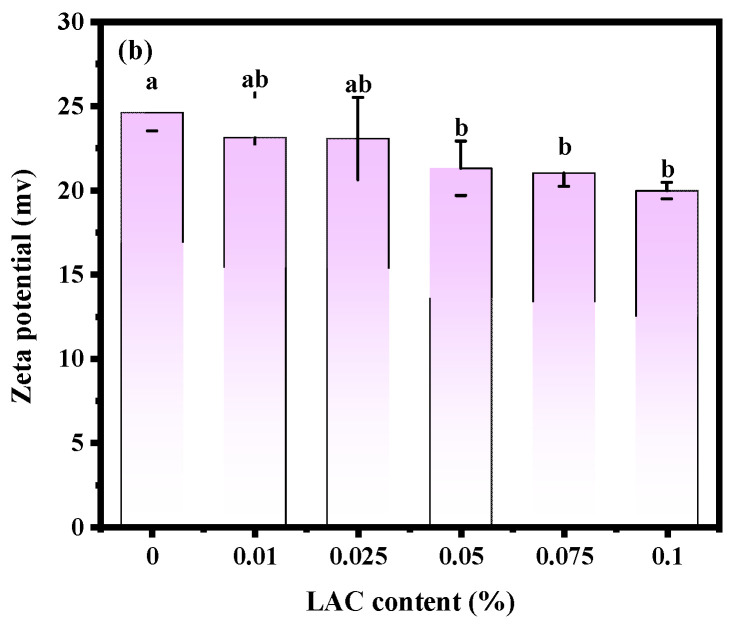
Effect of LAG on particle size distribution (**a**) and zeta potential (**b**) of soy yogurt. Different letters indicate that the values are significantly different (*p* < 0.05).

The stability of soy yogurt depends mainly on the particle size [36]. Results related to the particle size parameters of soy yogurt with different LAG concentrations are presented in Figure 4a. As the LAG concentration increases, the particle size of soy yogurt increased from 181.66 nm to 323.69 nm. The apparent particle size exhibited a monomodal distribution in all soy yogurt samples. Overall, the addition of LAG resulted in larger particles. The reason might be that higher polysaccharide addition level caused greater viscosity and weakened fluidity, thus forming particles with larger particle size. Additionally, zeta potential as another parameter was used to evaluate stability of the colloid system, indicating the electrostatic reactions between the particles in the suspensions. From Figure 4b, it can be observed that the zeta potential decreased from 24.61 mV to 19.97 mV as the LAG concentration increased. A similar trend was found in the observation of soy protein gel containing soy hull polysaccharides by Wang [37]. They ascribed the reason to decreasing electrostatic repulsion and interfacial film strength formed by soy protein isolates as the concentration of polysaccharides increased, which caused an increase in the size of droplets, and a decrease in the stability of emulsions.

### 3.5. Sensory Evaluation

**Figure 5:**
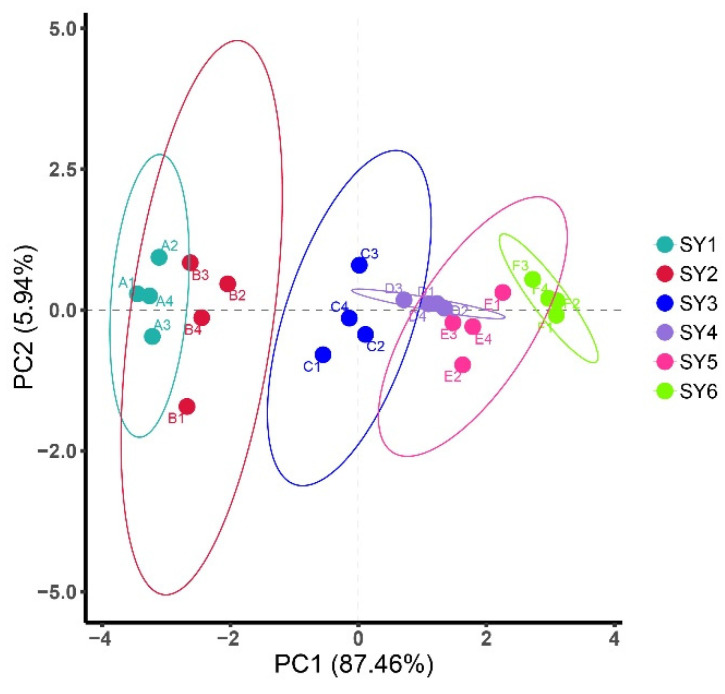
PCA plot showing the attribution of soy yogurt groups over LAG addition with respect to sensorial characteristics. (SY1, 2, 3, 4, 5, 6 with 0%, 0.01%, 0.025%, 0.05%, 0.075% and 0.1% LAG concentration, respectively).

The distribution of soy yogurt samples as a function of the two principal components extracted by PCA analysis was shown in Figure 5. PC1, which accounted for 87.46% of the variance, was mainly associated with elasticity, smell, chewiness, appearance, and taste. Meanwhile, for PC2, accounting for 5% is mainly related to color. Total variance of variables accounts for 93.4% presenting main sample information characteristics. Generally, all groups were distinctly differentiated. There are certain distances between the corresponding areas of sensory response values of soy yogurt added with LAG and native soy yogurt, suggesting that the addition of LAG made a difference to the sensory. When the concentration of LAG was 0.1%, soy yogurt exhibited the farthest distance, indicating the best sensory scores. 

## 4. Discussion

Soy yogurt, produced bacterial fermentation of soy milk, plays an important role in the present plant-based yogurt alternatives market owing to its high nutritional value and health aspects [1,38]. The further development of traditional soy yogurt production is limited by poor sensory experience, such as beany smell, insufficient acid production, rough taste, whey precipitation, and unstable curd. Hydrocolloids have been applied in soy yogurt to strengthen the connection between colloids and proteins, further enrich the taste, and improve the stability [39]. As a new type of microbial exopolysaccharide, LAG can achieve a good stability effect with a small amount added, but it is seldom used in the soy yogurt system. In this paper, we applied LAG in construction of three-dimensional composite network gel, investigating microscopic, macroscopic parameters. Moreover, sensorial properties of soy yogurt supplemented with LAG were also analyzed. Our results showed that LAG results in superior gelling property of soy yogurt, which was consistent with previous research, which indicated that yogurt with anionic polysaccharides was beneficial for enhancing water holding capacity, hardness, and rheological properties [27].

Physical properties (pH, viscosity, and water holding capacity) are important factors affecting the quality of soy yogurt products. During fermentation, yogurt culture (*Lactobacillus plantarum*) continuously produces lactic acid [40], which might lead to pH decrease. Poor stability may be attributed to precipitation of whey in the yogurt system [41]. In addition, water holding capacity reflects on the ability to retain water of yogurt [42]. The better the water holding capacity of yogurt is, the less whey is separated from yogurt by centrifugation [43]. For yogurt with LAG, 0.1% addition groups showed excellent water-holding ability, which is similar with that found in set yogurt. The polysaccharides with hydrophilicity absorb more water on the surface, enhancing gel rigidity [44]. Additionally, the electrostatic interaction with low pH between protein cluster with polysaccharides gradually strengthens, which forms a relatively stable compound, thus improving the characteristics of protein gel and contributing to WHC [45,46].

Whey precipitation generally brings out lower hardness and viscosity. Consequently, a texture analyzer was used to measure quality parameters including hardness, viscosity, chewiness, cohesive elasticity, and gel consistency. When the pH dropped to the isoelectric point of the protein (about 4.6), large amounts of negative charges carried by LAG attract positive charges, resulting in wrapping by protein micelles between LAG, preventing aggregation of protein micelles. The obtained product exhibited a general increase in hardness and viscosity. In addition, soybean proteins, which have been denatured and then solidified into gel in an acidic environment, formed a denser gel network structure and decreased the water precipitation rate of the product with the addition of LAG. Additionally, chewing showed a significant upward trend in a dose-dependent manner. The excellent gel properties might be attributed to formation of a stable and dense network structure, as well as renaturation of the triple helix of LAG, increasing crosslink of junction zones [47,48]. Tseng showed that the incorporation of LAG might contribute to strengthening electrostatic interaction between a glucuronic acid group with a negative charge of LAG and amino acid residue carried with a positive charge in soybean protein. Lorenzo reported that LAG could act as physical-fillings in the soybean protein network [49].

Soy yogurt is a viscoelastic material, so that the storage (G′) and the loss (G″) modulus were used to test rheological behavior, exploring the evolutionary process of the mixed gels. Heat and sterilization induced protein denaturation resulted in the lowest G’ values of soy milk. Accordingly, the network structure was denser with less porosity. With the concentration of LAG, G′ and G″ are obviously improved. When 0.1% LAG was added to the yogurt sample, the greatest G′ occurred, suggesting solid-like characteristics. The microstructure also suggests that application of LAG resulted in superior stability of soy yogurt. In the colloid system, LAG adsorbed on the soybean protein surface under the electrostatic interaction has thickened the adsorption layer and increased the charge number it could hold [50]. As the concentration of LAG goes up, the available binding sites on the protein surface were completely occupied, free LAG in a dispersed phase was influenced by a bridging effect which changes force balance, resulting in decrease of zeta potential. Additionally, the weak repulsive force has offset the thickened adsorption layer, leading to particle size increasing significantly.

In this study, the effect of LAG addition on soy yogurt acceptability was analyzed by sensory evaluation. The formation of relatively complex flavor composition of soy yogurt comes from native soybean milk, metabolite of bacterial growth as well as an interaction-produced new compound. Unpleasant perception such as beany flavor is eliminated due to fermentation treatment, accompanied with aroma-related chemicals including acetic acid, 2,3-butanedione, and 2,3-pentanedione 3-hydroxy-2-acetone [51]. Recent studies demonstrate that LAG could be used for *Lactobacillus plantarum* encapsulation to keep viability under low pH [52], leading to flavor substances’ accumulation, thus improving the sensory quality of yogurt. Our results showed that adding LAG at a 0.1% concentration received higher sensory acceptability, which was uniform in texture, with good flavor release and smooth taste. These also confirmed that LAG overcame the shortcoming of excessive addition of a single gel.

Our work applied LAG at a proper concentration for the improvement of physicochemical and structural properties of soy yogurt. It not only provides a new choice for overcoming the undesired instability of soy yogurt, but can also be applied for other plant-based yogurts.

## 5. Conclusions

To study the effect of LAG on the stability of a soy yogurt system, water holding capacity, texture, rheology, particle size and zeta potential were recorded. The addition of LAG at an appropriate concentration improved water holding capacity, gel stiffness and elasticity. Soy yogurts containing LAG were more acceptable in terms of sensory evaluation compared with the control group. Therefore, LAG is recommended as a stabilizer for soy yogurt. The addition of LAG to soy yogurt provided a new choice to improve stability during the production, transportation, and sale of soy yogurt.

## Figures and Tables

**Table 1:** Parameters of texture profile analysis (TPA) of soy yogurt with different LAG levels.

Level (*w*/*v* %)	Textural Parameters						
	Hardness 1 (N)	Hardness 2 (N)	Adhesiveness (N.s)	Cohesiveness (N.s)	Springiness (mm)	Gumminess (N)	Chewiness (mJ)
0	0.27 ± 0.04 ^c^	0.25 ± 0.04 ^c^	0.45 ± 0.17 ^a^	0.68 ± 0.06 ^a^	7.40 ± 4.48 ^a^	0.19 ± 0.03 ^c^	1.30 ± 0.72 ^c^
0.01	0.33 ± 0.05 ^c^	0.26 ± 0.05 ^c^	0.48 ± 0.25 ^a^	0.61 ± 0.26 ^a^	6.27 ± 5.21 ^a^	0.20 ± 0.06 ^c^	1.39 ± 1.38 ^c^
0.025	0.59 ± 0.07 ^c^	0.47 ± 0.11 ^c^	0.47 ± 0.22 ^a^	0.47 ± 0.18 ^a^	9.55 ± 0.76 ^a^	0.28 ± 0.12 ^c^	2.67 ± 1.17 ^bc^
0.05	1.11 ± 0.34 ^b^	0.87 ± 0.09 ^b^	0.48 ± 0.31 ^a^	0.59 ± 0.21 ^a^	9.04 ± 1.62 ^a^	0.62 ± 0.14 ^b^	5.66 ± 1.99 ^b^
0.075	2.11 ± 0.37 ^a^	1.81 ± 0.26 ^a^	0.54 ± 0.23 ^a^	0.63 ± 0.14 ^a^	9.72 ± 0.44 ^a^	1.30 ± 0.20 ^a^	12.69 ± 2.43 ^a^
0.1	2.34 ± 0.16 ^a^	1.95 ± 0.08 ^a^	0.62 ± 0.11 ^a^	0.60 ± 0.01 ^a^	9.97 ± 0.03 ^a^	1.41 ± 0.10 ^a^	14.00 ± 1.01 ^a^

Means with different superscripts in each column are significantly different (*p* < 0.001). All values are the means ± standard deviations of three replicates.

## Data Availability

All persons included have agreed to confirm.

## Supplementary Materials

The following supporting information can be downloaded at: https://www.mdpi.com/article/10.3390/gels8070453/s1. Figure S1. The stress curve of the soy yogurts supplemented with different concentrations of LAG (0%, 0.01%, 0.025%, 0.05%, 0.075%, and 0.1%). Figure S2. The strain sweep curve of the soy yogurts supplemented with different concentrations of LAG (0%,0.01%, 0.025%, 0.05%, 0.075%, and 0.1%).
