# Supplementary material for: Physicochemical, Textural, and Sensorial Properties of Soy Yogurt as Affected by Addition of Low Acyl Gellan Gum
Source: Gels. 2022 Jul 20;8(7):453. doi: 10.3390/gels8070453 (PMC9318443; doi:10.3390/gels8070453)
Supplement: Supplementary file 1 [file gels-08-00453-s001.zip › gels-1816900-supplementary.pdf]

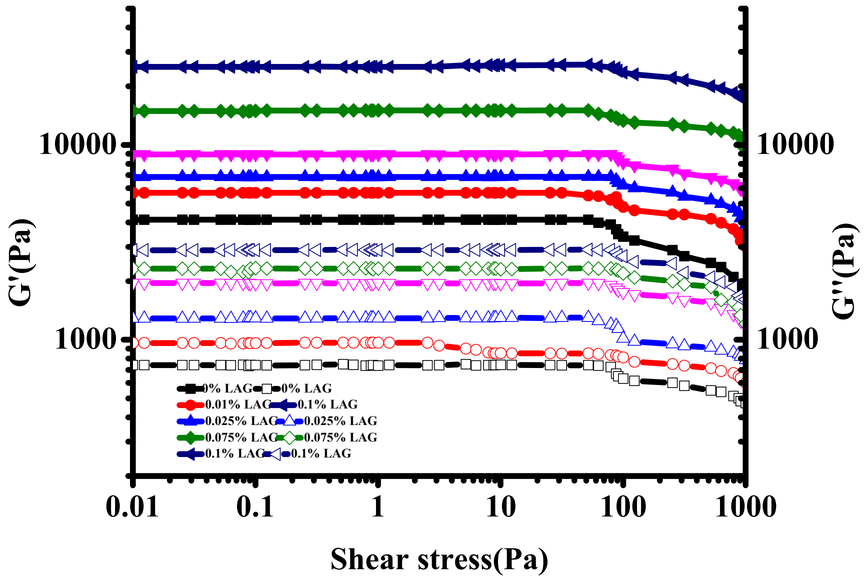

Figure S1. The stress curve of the soy yogurts supplemented with different concentrations of LAG (0%, 0.01%, 0.025%, 0.05%, 0.075%, and 0.1%).

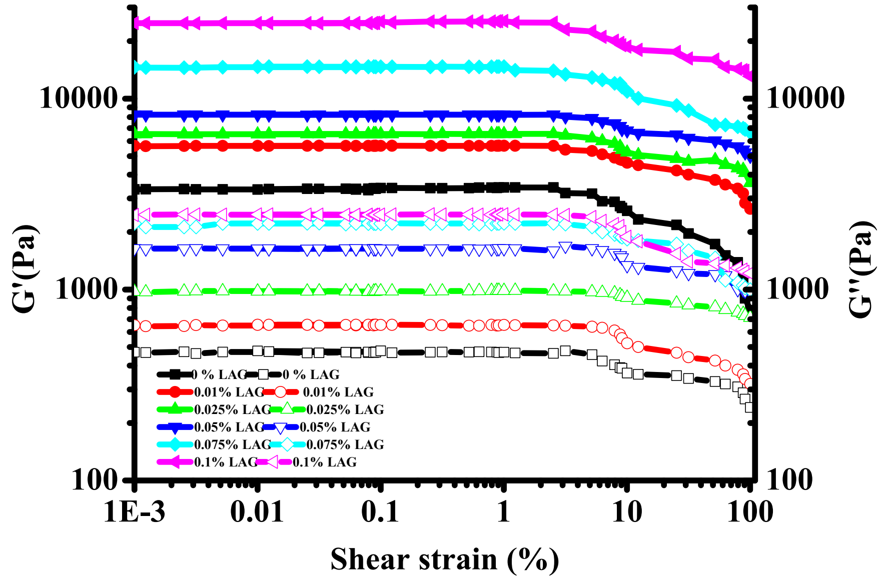

Figure S2. The strain sweep curve of the soy yogurts supplemented with different concentrations of LAG (0%,0.01%, 0.025%, 0.05%, 0.075%, and 0.1%).
